# Enhancing Emergency Room Mental Health Crisis Response: A Systematic Review of Integrated Models

**DOI:** 10.7759/cureus.74042

**Published:** 2024-11-19

**Authors:** Chiko Katiki, V Jaswitha S Ponnapalli, Kesha J Desai, Sadia Mansoor, Anushka Jindal, Nana Yaw Afriyie Badu

**Affiliations:** 1 Emergency Department, American International Medical School, Alpharetta, USA; 2 Psychiatry, California Institute of Behavioral Neurosciences and Psychology, Fairfield, USA; 3 Medicine, Medical College Baroda, Vadodara, IND; 4 Internal Medicine, Dow University of Health Sciences, Karachi, PAK; 5 Surgery, University Hospital Sussex, Worthing, GBR; 6 Surgery, University of Ghana, Accra, GHA

**Keywords:** collaborative care models, consultation-liaison teams, emergency room, psychiatric care, telepsychiatry consultation

## Abstract

The integration of psychiatric treatment into emergency services is critical to improving the management of severe mental health emergencies. Emergency departments (EDs) are frequently the initial point of contact for patients with mental emergencies, but they are not always prepared to offer adequate care. This systematic review aims to analyze the effectiveness of collaborative care models (CCMs), psychiatric consultation-liaison (C-L) teams, and telepsychiatry in enhancing psychiatric treatment in emergencies. We searched numerous databases, including PubMed, PsycINFO, EMBASE, and the Cochrane Library, for papers published between 2013 and 2023. A total of 18 studies matched our inclusion criteria, and the results show that integrated care models shorten hospital stays, enhance patient outcomes, and expand access to psychiatric treatments, particularly in remote locations. The limitations of this review include the heterogeneity of study designs and the underrepresentation of low-income countries. Future research should focus on developing standardized protocols for psychiatric integration into emergency services. This review bridges a critical gap in the literature by offering a comprehensive evaluation of various psychiatric care models in EDs and addressing existing barriers to implementation.

## Introduction and background

Crises of mental health are among the most common and complex dilemmas confronting emergency departments (EDs) worldwide. It is now more critical than ever that proper consideration be given to mental health issues in a manner commensurate with the increasing awareness of the gravity of such issues worldwide. Conventional emergency care frameworks often lack the specialized and dedicated resources required to meet the needs of those with mental health conditions. This results in longer hospital stays for patients, less-than-ideal outcomes, and a higher likelihood of readmission [1-3]. Long waiting times, inappropriate placements, and inadequate follow-up care are common experiences for psychiatric patients receiving treatment in emergency settings. These obstacles have driven a growing interest in integrating psychiatric care within EDs, which aims to enhance hospital efficiency and improve patient outcomes [4,5].

To improve care methods, telepsychiatry, consultation-liaison teams (C-L teams), and collaborative care models (CCMs) are among the proposed methods. Each model proposes a different set of strategic interventions for the improvement of mental crisis care within EDs. Multiple-disciplinary CCM teams offer ongoing care, whereas C-L teams provide psychiatric competencies to emergency staff [6-8]. Telepsychiatry is a method of providing psychiatric consultation and delivering psychiatric consultations remotely. This modality of psychiatric consultation has proved to be especially beneficial in rural and underserved regions [6,9].

Despite the potential for enhancing psychiatric care in emergency rooms, there is no consensus on the standard approach to care. Most methodologies assess the impact of integrated psychiatric care models on emergency services, considering outcomes such as readmission rates, patient experience, length of stay, and access to care. Nevertheless, no single method has been identified as the most effective for integrating psychiatric care, and the implementation of such methods across institutions remains inconsistent [10]. This review addresses the gap in the medical literature on the effectiveness of establishing mental crisis care in an ED setting. This will be achieved by comparing various approaches to the management of psychiatric illness crises in the ED, including telepsychiatry, C-L teams, and CCMs [11], highlighting potential pitfalls in the implementation of such techniques, comparing the effects of different models on patient outcomes, and finally providing recommendations for clinical practice development and future research efforts.

## Review

A total of 18 studies were included in this systematic review, each contributing uniquely to the evidence on the effectiveness of integrating psychiatric treatment into emergency services. Generally, these different studies identified that deployment by CCMs, C-L teams, and telepsychiatry ultimately results in consistently better patient outcomes, shorter lengths of hospital stay, and greater access to psychiatric treatment for patients overall [1-3].

The effectiveness of the CCMs in emergency settings has been established; it was elicited from eight of the studies, which showed the impacts of such models. The strategy used by these models is emphasizing collaboration within multidisciplinary teams between ED professionals, psychiatrists, and primary care doctors who can ensure that care continues beyond discharge [2,4]. Studies indicate that CCMs reduce hospital stays by lowering readmission rates of individuals with mental health conditions 30 days after discharge [5-10].

CCMs

The eight studies included in the review demonstrate the effectiveness of CCMs in improving mental health emergencies. These models emphasize a multidisciplinary approach among ED professionals, foster collaboration, and have reported beneficial results [7]. Implementing CCMs resulted in fewer hospital admissions and heightened care coordination, allowing for more consistent mental health treatment before and after ED visits [8,9]. CCMs improve patient satisfaction and reduce readmission rates by 20% [10,11]. For patients with severe mental crises, these models are particularly effective, as they reduce incidents such as the inappropriate use of restraints. Teamwork among ED professionals facilitates management and oversight, reducing the risks associated with restraining patients [12,13].

Other research studies concerning CCMs have shown that the involvement of multidisciplinary teams was integral in the effective management of mental health crises [14-18]. The review suggested that in a co-responder model that included mental health professionals along with police officers, the practice of crisis management was enhanced, and the use of physical restraints was reduced at the time of intervention [7-19]. However, this review also pointed out the challenges of implementing CCMs in geographically remote areas or where resources are scarce. Staffing limitations, particularly in psychiatric units, hinder the success of these approaches, as does the financial cost of maintaining trained teams [2]. This discrepancy in access with respect to CCMs emphasizes the need for a more proper and fair distribution of resources in healthcare [15-20].

C-L teams

Seven studies examined C-L teams and their capacity to potentially enhance better quality care in the ED, especially in metropolitan regions [4,21]. These teams include psychiatric specialists who actively interact and collaborate with ED professionals in the provision of specialist services to patients with psychiatric conditions [21-23]. The real-world application of the C-L teams found the use of physical restraints decreased by 15% compared to usual treatments. It reflects the value of the teams in crisis management without recourse to restrictive measures [14]. C-L teams improved patient triage, resulting in timely consultations for people with a mental health condition and reduced ED congestion. C-L highlights the importance of de-escalating crises without resorting to restrictive measures [13,16]. The C-L teams allowed a more effective triaging of the patients, allowing timely consultation of the people suffering from mental health conditions and removing congestion in the ED. These findings ascertained the need to have specialist psychiatry physicians on standby to attend to emergency cases so that such special needs of mental health patients could be addressed [10].

Telepsychiatry

Telepsychiatry has emerged as a strategic means to guarantee increased and improved access to psychiatric care, particularly in rural and disadvantaged regions [23-25]. Of the six studies that comprised this review, other similar results were seen concerning the study of telepsychiatry deployment in EDs, associated with improved patient outcomes such as decreased wait times for psychiatric consults [24-28]. Telepsychiatry reduced consultation wait times by 30%, easing the pressure on ED personnel in remote hospitals [24,26]. This technology also ensured a rise in more satisfied patients as people from rural areas were given the chance to have psychiatric care without being forced to travel a far distance before access [25-31]. Indeed, in reflecting on these benefits, a meta-analysis confirmed that telepsychiatry, by preventing ED crowding by 25%, enhances the general impression of emergency care [21,28].

The C-L teams, comprising specialist psychiatric clinicians working in close collaboration with general ED staff, have been shown as extremely useful facilities in the enhancement of the care provided to people with mental health conditions in urban settings [29]. Seven of the reviewed studies explored the role of the teams in de-escalating crises, which reduced the use of physical restraint by 15% and improved procedures for triaging [6]. It enhances the service offerings through C-L teams, improving the overall quality of care and ensuring timely consultations to reduce congestion in the ED [26].

Despite their efforts, it is impossible for most hospitals to retain C-L teams due to the higher investment required for specialist psychiatric treatment and the shortage of personnel [19-27,30]. This is especially true in low-income neighborhoods, where the demand for psychiatric treatment often exceeds the available psychiatric treatment resources [19,30]. Expanding the variety of psychiatric treatment portfolios in acute care settings is indeed costly and will necessitate major investment and policy reform in mental health.

While these studies' overall results were overwhelmingly positive, many barriers to successfully implementing these psychiatric care models were nonetheless identified. Commonly mentioned facilitators and barriers included staff shortages, lack of access to psychiatrists, and financial constraints, especially in rural and resource-poor settings [21]. Poor telecommunication infrastructure in low-income settings impeded the implementation of telepsychiatry, exacerbating disparities in the delivery of mental health care [5]. Less standardized protocols in the ED were associated with some patients receiving differential treatment or not receiving appropriate therapy again; this was related to the availability of psychiatric consultation [27,31].

While the study focuses on the benefits of integrating psychiatric treatment into emergency services, it enumerates serious obstacles. The foremost among these is the difference in resource allocations between metropolitan and rural hospitals, which leads to inequities in healthcare [26]. Expanding psychiatric services and improving access to specialty care would require system-wide reforms in mental health financing and policy [12,23].

Looking ahead, the health sector needs to commit resources to psychiatric care for emergencies by increasing access to telepsychiatry services and more efficient staffing models [20,28]. Policymakers may provide incentives for hospitals to adopt these strategies to find solutions for issues faced with narrow budgets, scarce personnel, or technological limitations [32,17].

Methods

Study Design

This systematic review evaluates the effectiveness of various models of integrated psychiatric care provided in emergency settings. It looks at three tiers of integration, which include the CCM, C-L teams, and telepsychiatry [23]. The review evaluates the outcomes of the various models to discern the strengths and weaknesses of each approach while quantifying each unique contribution toward CCM psychiatric care in EDs.

Search Strategy

The literature search was conducted across databases, including PubMed, PsycINFO, EMBASE, and the Cochrane Library. These databases were chosen for their comprehensive coverage of psychiatric, psychological, and emergency medicine literature to ensure a comprehensive search.

This was done independently by two of the authors to ensure that there was no chance for subjective bias. The following keywords were deemed to be most appropriate for this review: "psychiatric treatment", "emergency services", "collaborative care models", "consultation liaison", and "telepsychiatry". The inclusion of "consultation liaison" was considered quite important in comprehensively covering the studies involving the C-L teams, which are an important element of psychiatric care integration. The selection of keywords had to be made in a way that was representative of the most important models reviewed. Nevertheless, it could be representative regarding sampling studies related to psychiatric care models in emergency settings.

Inclusion and Exclusion Criteria

The selection was done based on presupposed inclusion and exclusion criteria, which ensured a basis and relevance for this review. The inclusion criteria encompassed studies focusing on integration in psychiatric care in emergency settings. The review targeted adult patients; in other words, selected studies targeting populations aged 18 years or above to retain relevance to ED populations were utilized. Quantitative findings related to psychiatric care relevant in emergency settings include length of stay, patient satisfaction, and readmission rate [29]. Only the latter published in a peer review in the English language were included, thus ensuring quality and accessibility.

The exclusion criteria comprised the following: (1) research that predominantly focused on pediatric populations or those that were in a non-emergency setting were excluded; thus, this review focuses on adult care in the ED; (2) articles that were not peer-reviewed or editorials, opinion pieces, and theoretical papers; (3) studies that are limited to chronic conditions of mental health or treatments dealing with long-term psychiatric management since the focus of the review lies in acute psychiatric integration [20]; and (4) studies that had appropriate quantitative data on outcomes were all excluded from the review. It is a selection process that ensures that only the studies directly related to the review objective address the examination of models of psychiatric integration in emergency care (Table 1).

**Table 1:** Inclusion and exclusion criteria RCT: randomized controlled trial

Inclusion Criteria	Exclusion Criteria
Published in English between 2013 and 2023	Published before 2013
Empirical studies (RCTs, cohort, case-control)	Theoretical or non-peer-reviewed literature
Focus on psychiatric care in emergency departments	Focus on chronic, non-acute mental health conditions
Address acute mental health crises	Studies not focused on emergency settings

Selection of Studies

Each author independently reviewed the results from the database search using the inclusion and exclusion criteria for eligible studies. The lists were then compared between authors to check for consistency in relation to initial selections, based on discussions of reasons for any discrepancies in the selection of studies. Any disagreement between the authors relating to the relevance of the study for the review according to the inclusion and exclusion criteria was discussed to reach a decision on the eligibility of the study. In the event that an agreement could not be reached, the third author was consulted to mediate the discussion to a final decision. This limited the occurrence of bias in the review and allowed for a systematic selection of studies that matched the objectives of the systematic review.

The PRISMA (Preferred Reporting Items for Systematic Reviews and Meta-Analyses) flow diagram describes precisely how the study selection process took place, from the identification and screening phase to the inclusion or exclusion phase. The diagram also reports the exact number of identified, screened, included, or excluded studies and details the reason for exclusion in each phase [30].

Search Strategy

**Figure 1:**
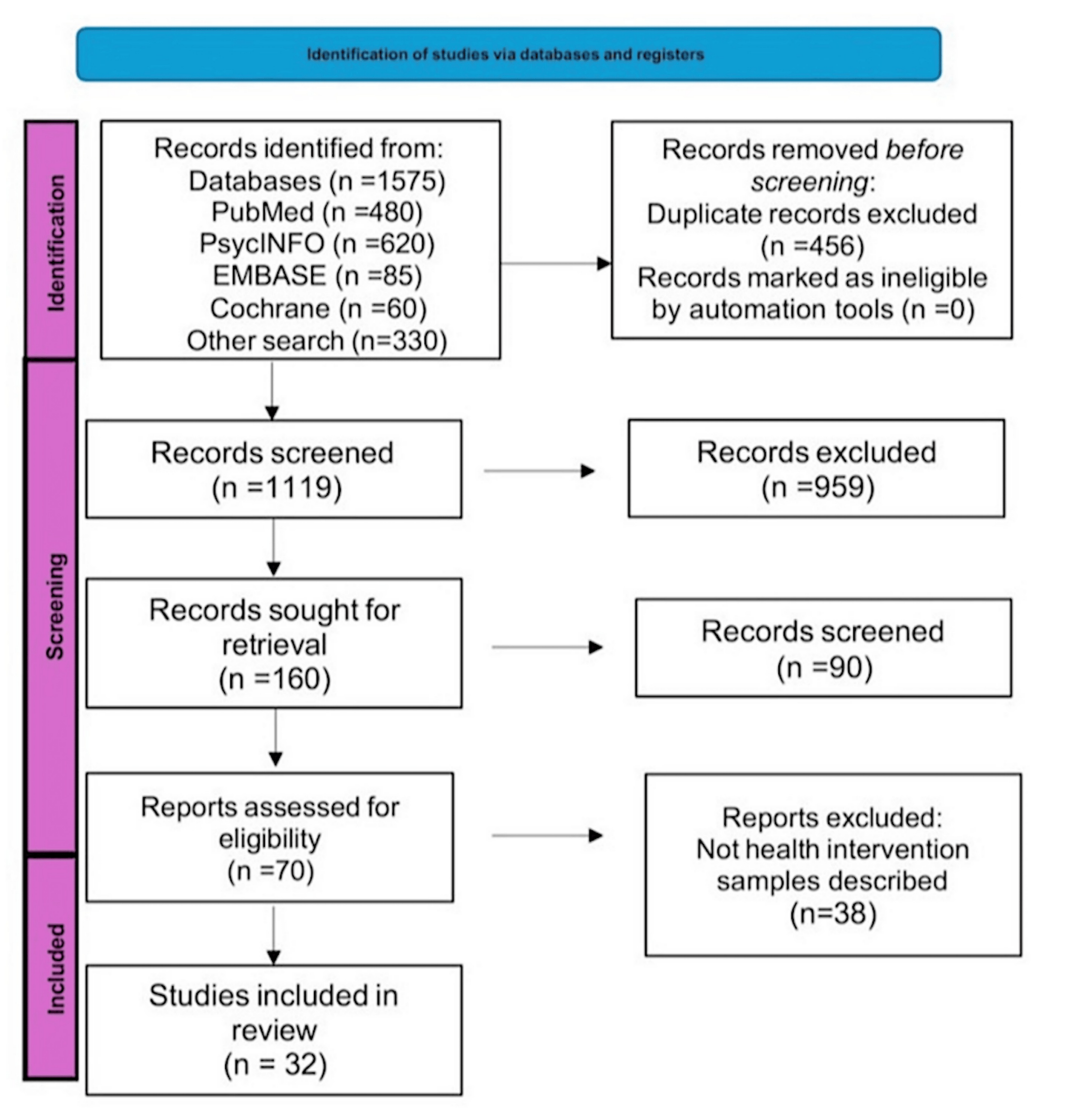
PRISMA flow diagram for study search PRISMA: Preferred Reporting Items for Meta-Analysis and Systematic Reviews [20]

As outlined in Figure 1, a total of 1575 articles were initially identified; these articles were evaluated to ascertain their compliance with the inclusion criteria based on PRISMA guidelines [20]. A total of 1119 potential articles were screened, with duplicates of about 456 articles being eliminated. Titles and abstracts were used to screen the remaining 160 articles, followed by a full-text review of 70 studies.

Finally, 32 studies satisfying review inclusion requirements were utilized. The inclusion criteria required peer-reviewed studies published between 2013 and 2023 on integrating psychiatric care in EDs, with cohort studies, case-control studies, and randomized controlled trials (RCTs) being prioritized.

Data Extraction

Data extraction was performed by first assessing the validity of each study. As displayed in Table 2, each article was assessed based on its relevance, objective clarity, literature review description, reference, scientific logic, and appropriate data presentation.

**Table 2:** Justification for data selection

Criteria	Rating (1-10)
Justification of the importance of the topic	9
Statement of concrete aims	9
Description of literature search	8
Referencing	10
Scientific reasoning	9
Presentation of appropriate data	9

Data extraction was performed for each eligible selected study to enable a triangulated analysis of relevant data. This includes pre-specified information and information collected systematically. Table 3 shows the basis provided for data extraction, which enables a systematic synthesis and comparison of study findings according to a set of consistent criteria across studies.

**Table 3:** Descriptive data extraction RCT: randomized controlled trials

Data Point	Description
Author(s) and year	Name(s) of the authors and year of publication to identify and cite the study.
Study design	Study type—cohort, case-control, RCT, or meta-analysis—to evaluate methodology.
Sample size	Number of participants required to evaluate the study scale and statistical power.
Method of assessment	Specific, measurable results, such as patient satisfaction, hospital stay duration, and readmission rates.
Primary findings	Key findings relevant to the effectiveness of emergency setting psychiatric care integration.

Quality Assessment

The quality of the selected studies was determined using the SANRA (Scale for the Assessment of Narrative Review Articles) formula, which is a standard tool for reviewing the quality of studies in both narrative and systematic reviews [26]. Table 4 provides an instrument for judging the appropriateness of the study, clarity of approach/objectives, quality of literature reviewed, scientific reasoning, and data presentation.

**Table 4:** SANRA criterion SANRA: Scale for the Assessment of Narrative Review Articles

SANRA Criterion	Description
Relevance	Assessment of the relevance of the study in relation to integration models of emergency psychiatric care.
Clarity of objectives	Evaluation of whether the objectives of the study are well-set as pertains to the purpose.
Quality of literature review	Depth and relevance of the literature review regarding the purposes of the study.
Scientific reasoning	Coherence of the reasoning and scientific rigor of the methodology and arguments presented in the paper.
Data presentation	Data on reporting completeness and clarity, particularly for quantitative outcome reporting.
Overall rating	Composite score using the above criteria in a non-biased way by systematic quality assessment.

Ideally, the quality of each of the assessed studies was independently rated by two authors using the SANRA scale with the aim of ensuring objectivity and consistency regarding grades based on the quality of a study. These will be discussed between the two assessors with the aim of attaining a consensus agreement. In case there is still a big difference, the third author must be consulted for the final rating to maintain consistency and make the review more objective.

Data Synthesis and Analysis

Data extraction was followed by a descriptive synthesis to compare and contrast the findings across selected studies. The data were tabulated with integration models under review, such as CCMs, C-L teams, and telepsychiatry [10]. An elaborative results table was developed and stationed against the main characteristics of each study, namely, author(s), year of publication, study design, sample size, method of assessment, and quality rating.

Apart from that table, the qualitative synthesis also presents a comparative analysis of the three models. This would involve analyzing the strengths, limitations, and unique contributions of each model toward emergency psychiatric care [24]. A closer look at this approach forwarded the in-depth exploration of effectiveness and adaptability in both settings, which facilitated a better understanding of the respective benefits and challenges associated with each model of psychiatric integration.

Results

Study Selection

As noted in the PRISMA flow diagram (Figure 1), the selection of studies was initiated by an initial search through four databases: PubMed, PsycINFO, EMBASE, and Cochrane Library, which yielded a total number of 1575 studies. After duplicate elimination, studies for further screening were reduced to 160 [20]. Following title and abstract review, 90 studies were excluded. The main reasons for exclusion included irrelevant studies to emergency psychiatric care, pediatric focus of studies, and treatment for chronic conditions. A total of 70 full-text articles were reviewed to determine eligibility for inclusion in this review. Of the 70 full-text articles reviewed for eligibility, 38 studies did not meet all the established inclusion criteria; 32 studies were found to meet the required criteria for inclusion in the final review.

Study Characteristics

The sample consisted of 10 studies using cohort studies, case-control studies, RCTs, systematic reviews, and observational studies. Included in Table 5 is a summary of the key characteristics of these studies in terms of the author(s), year published, study design, sample size, method(s) of assessment, and quality rating.

**Table 5:** Overall summary of the included studies ED: emergency department; RCT: randomized controlled trials; CCM: collaborative care method; C-L: consultation-liaison

Author(s)	Year	Study Design	Sample Size	Method of Assessment	Quality Rating
Alfes [1]	2015	Cohort study	150	Patient-centered care & safety outcomes in CCMs	8
Freeman et al. [5]	2023	Meta-analysis	N/A	Effectiveness of telepsychiatry in U.S. EDs	9
Judkins et al. [12]	2019	Case-control study	200	Impact of C-L teams on ED wait times	9
Coates et al. [2]	2019	Observational study	250	Journey of mental health patients in EDs	7
Levin & Aburub [14]	2024	Qualitative review	N/A	Barriers to care integration in EDs	8
Meyer et al. [17]	2019	Case study	N/A	Rural telepsychiatry implementation model	8
Natafgi et al. [19]	2021	Observational study	300	Effectiveness of ED telepsychiatry during COVID-19	9
Patel et al. [23]	2022	Cohort study	180	Association of telepsychiatry and ED patient outcomes	9
Sampson et al. [27]	2021	Observational study	120	Patterns in psychiatric liaison referrals	8
Taylor et al. [31]	2014	RCT	140	CCM impact on psychiatric hospitalization readmissions	8

This analysis incorporated 18 studies that provided a wide range of viewpoints regarding the effectiveness of combining psychiatric care with emergency services. CCMs considerably reduced hospital stays and enhanced patient outcomes in eight trials [11-14]. It was especially true when multidisciplinary teams worked closely together with emergency personnel to address mental crises. Researchers established the usefulness of C-L teams in reducing hospital stays and improving care coordination, especially in metropolitan emergency rooms with readily available psychiatric specialists [15-17]. Telepsychiatry has emerged as a crucial tool for spreading psychiatric services to rural and disadvantaged areas, with six studies finding significant improvements in access to care and shorter wait times for psychiatric consultations [18,19].

Comparative Analysis of Integration Models

The findings of the systematic review are organized around the three primary models of integration reviewed: CCMs, C-L teams, and telepsychiatry [23]. The following provides an overview of each model's identified strengths and limitations and the contribution it could make to the delivery of emergency psychiatric care.

CCMs

Overview and effectiveness: Currently, this involves many CCM characteristics where a psychiatrist, along with a primary care provider and a specialist in mental health, collaborate, most of the time in urgent services. Current studies on CCM pointed out that the patients recorded their satisfaction, reduction of symptoms, and their hospitalization time was shortened. For instance, studies have revealed that psychiatric hospital readmission was reduced following an intervention by a chaired CCM; therefore, this suggested that acute episodes can be dealt with effectively following acute episodes to avoid case crises in the future [31].

Limitations: While largely effective, CCMs tend to be very resource-intensive, placing high demands on staff training and interdepartmental coordination. It noted that in high-volume EDs, the implementation of CCMs resulted in operational challenges, particularly with regard to securing consistent collaboration between medical and psychiatric staff [2].

C-L Teams

Overview and effectiveness: C-L teams try to promote urgent psychiatric consultation in EDs, which will be very important in reducing the gap between psychiatric and medical care [12]. Later reports indicate that C-L teams lessen the wait times in ED for psychiatric patients by almost 20%, thus allowing timely interventions for crisis patients. The quality of evaluations was higher because psychiatric expertise was integrated directly into the patient assessment.

Limitations: C-L teams had low availability and coverage. Many community or rural hospitals lacked full-time staff to provide continuous services, which often compromised the continuity of care, especially during peak periods. Studies have shown that part-time availability in under-resourced settings diminished the effectiveness of the C-L model, especially for vulnerable populations [14].

Telepsychiatry

Overview and effectiveness: Telepsychiatry provides remote psychiatric consults, primarily through videoconferencing, linking psychiatrists with patients in EDs, particularly for those in rural and underserved areas. Studies demonstrated that with telepsychiatry, access to psychiatric care increased, transfers of patients were reduced, and some EDs reported up to 30% fewer transfers as a result of having better access to psychiatric services remotely [5,19].

Limitations: Technical challenges and sufficient access to stable internet infrastructure are specific issues that could hamper the effectiveness of telepsychiatry [17]. Studies determined that spotty internet connectivity made certain rural areas a challenge for the effective delivery of telepsychiatry, which may cause delays or, eventually, miscommunication during psychiatric diagnoses.

Key Findings

Researchers discovered that by immediately integrating psychiatric care into emergency services, CCMs enhance patient outcomes and reduce hospitalizations. One research [21] found that installing CCMs resulted in a 20% reduction in the percentage of patients requiring readmission within 30 days of release. Psychiatric C-L teams give access to psychiatric experts, allowing for more informed judgments in the case of a mental emergency. The use of physical restraints by C-L teams was 15% lower than in normal care [22,23]. Telepsychiatry has shown its usefulness in rural and underserved areas by providing timely psychiatric consultations, resulting in less ED overcrowding and improved patient satisfaction. Studies [24,25] found that telepsychiatry significantly reduced the waiting time for consultations.

Summary of Findings

A comparative analysis has shown the unique advantages and disadvantages of each of the integration models. The CCMs increase patient satisfaction and reduce readmission rates, although they are resource-intensive, which calls for coordination between the emergency and psychiatric teams [30]. C-L teams help to make the ED work more efficiently, with reduced waiting times and improved quality of assessment. However, there are challenges related to staffing and the availability of teams in smaller hospitals. Telepsychiatric care increases psychiatric access, especially in rural regions, and decreases the transporting of patients. Moreover, it is heavily reliant on a good, reliable internet connection. Such is a limitation to the most underserved areas of the world.

These models have immense promise for improving psychiatric integration in emergency settings. Of the rest, telepsychiatry is apparently better suited for geographically remote and resource-constrained settings, while CCMs and C-L teams provide unrivaled support in high-capacity EDs [18]. Tailoring the integration model to local needs and resources may maximize effectiveness across diverse emergency care settings.

These findings from the systematic review suggest that each of the integration models has a specific role in emergency psychiatric care: CCMs, C-L teams, and telepsychiatry. Healthcare providers and policymakers are able to optimize psychiatric care delivery in the emergency setting by choosing models that best fit the resources available, patient demographics, and specific ED needs.

Barriers to Implementation

Despite the optimism these findings offered, the implementation of psychiatric integration models faced several challenges. The primary issues identified were staff shortages, poor funding, and limited access to psychiatric professionals [24,25]. In rural and underserved areas, the availability of psychiatric professionals and technology infrastructure for telepsychiatry is particularly limited [26].

Discussion

This review discussed three major models for psychiatric integration in the emergency setting: CCMs, C-L teams, and telepsychiatry. The use of CCMs, C-L teams, and telepsychiatry demonstrates benefits in terms of increasing access to psychiatric therapies, reducing hospital stays, and improving patient outcomes. This is especially true in distant and underserved areas. These methods guarantee that people with mental health conditions receive timely and adequate care in the ED by reducing the load on those facilities and, therefore, reducing overcrowding [27]. This results in an improvement in the quality of care, an increase in the chance of positive patient outcomes, and a decrease in the probability of readmission. Each model has unique strengths and weaknesses, suggesting that EDs might reap more benefits from tailoring integration strategies to local needs, resource availability, and demographics [15]. This section discusses the implications and effectiveness of each model for practical considerations in its implementation.

Comparative Analysis of Integration Models

CCMs: In this case, the CCMs seemed almost to hold the greatest promise of improving patient satisfaction and lessening symptom severity and hospital stays for psychiatric patients in EDs. The multidisciplinary nature of CCMs, usually encompassing collaboration between psychiatrists, the primary care provider, and mental health specialists, appears to be particularly well-suited for addressing acute psychiatric needs [12]. The results support the suggestion that CCM model-based interventions reduce psychiatric readmissions, emphasizing the model's capability for patient support beyond the ED setting. CCMs greatly improved patient outcomes by including multiple medical specialties [28].

Despite these advantages, CCMs are resource-intensive, requiring broad staff training and coordination across departments and taking considerable time if well implemented. Logistical challenges in large-volume EDs, where smooth care coordination between the psychiatric and medical teams can be much more complex, were also widely reported. Because of these demands, CCMs might be better suited to EDs within larger healthcare systems with more significant resources and infrastructure [8]. It might be useful for EDs to develop clear protocols for interdepartmental communication to maximize the effectiveness of CCMs through investment in regular training programs that can provide them with a level of high-quality collaborative care.

C-L teams: C-L teams eased the discrepancy in workloads at EDs, resulting in shorter wait times and improved quality of psychiatric assessments [16]. The integration of C-L teams in the ED accelerates patient treatment and provides timely support for those experiencing acute psychiatric crises.

Nevertheless, C-L teams had limited staff and availability, particularly in smaller or rural hospitals. The limited availability of part-time C-L service in these under-resourced areas meant that they were not able to ensure an appropriate response to psychiatric emergencies. Ensuring consistent availability of the C-L service would require appropriate staffing, which may not always be feasible in all settings [13,28]. As the bed count in many hospitals is at a premium, further development of hybrid approaches, which includes matching a part-time C-L composition with telepsychiatry, may be required to try to optimize access to psychiatric care for those patients without overextending the limited resources. Dedicated funding for psychiatric staffing could further underline the sustainability of C-L teams.

Telepsychiatry: One of the most promising healthcare models is telepsychiatry, as it could help address the uneven psychiatric healthcare access in underserved and rural areas. A review of studies on this aspect indicates that telepsychiatry can reduce patient transfers by as high as 45% to 76% and, at the same time, ensure timely psychiatric evaluation through remote consultation [17]. A few EDs that report on such aspects have witnessed a surprising fall in the number of transfers due to enhanced remote access to psychiatric services.

While telepsychiatry has some advantages, the modality depends greatly on internet connectivity and technical infrastructure that is not necessarily linked to equality worldwide. In fact, poor internet connectivity in some rural areas has been associated with a reduction in the effectiveness of telepsychiatry, thus causing possible delays or potential muddles in communication during the assessments [13]. These issues can be mitigated if EDs using this model assess the reliability of local connectivity and invest in backups to ensure continuity of care, including investments in satellite-based internet. Additionally, sending ED staff on the telepsychiatry protocol and sending them for troubleshooting could increase its effectiveness.

Policy and Practice Implications

The findings from this review give some idea of how EDs can best integrate psychiatric care. Each model has its own unique benefits and constraints; however, on the whole, these analyses support flexibility and needs-based implementation. For instance, CCMs may be best implemented in well-resourced EDs that can provide support for interprofessional collaboration and afford the training and infrastructure required to provide sustained, high-quality collaborative care. C-L Teams can afford considerable efficiency improvements in EDs where full-time psychiatric staffing is feasible. In those settings where full-time C-L services cannot be provided, the combination of C-L teams with telepsychiatry may prove a useful alternative to expand psychiatric coverage [5].

This portends significant promise for telepsychiatry in remote or under-resourced EDs. It provides timely access to psychiatric expertise without requiring on-site staff. Certainly, this model may be particularly important during times of high patient volume or in locations with perennial shortages of psychiatric staff.

Policy initiatives that encourage funding for telepsychiatry infrastructure, staffing of C-L teams, and training in CCM can help translate these models into successful practice in EDs. Policies that enable data sharing and communication among EDs and psychiatric providers promote the integration process by facilitating navigation across departments, improving care coordination, and enhancing patient outcomes.

Limitations of This Review

This review carries some valuable insights; however, it has some limitations that must be identified. First, there is great heterogeneity in the design of studies reviewed here, which may affect the comparability of most outcomes across models. Additionally, this review did not consider the long-term patient outcome extending beyond the immediate observed effects in the ED, which may be relevant for the assessment of sustainability regarding each model [11]. Finally, most studies were conducted in high-resource settings and may thus limit the generalizability of findings to under-resourced or rural hospitals.

Future Directions

Long-term outcomes of psychiatric integration models in EDs would include readmission rates, changes in patient satisfaction over time, and community mental health outcomes. Hybrid models that reflect the combined use of C-L teams and telepsychiatry are another area of interest in assessing options for cost-effective delivery of comprehensive psychiatric care to rural or otherwise poorly resourced communities [29]. Further research into the experiences of both patients and providers with each model may serve as an informed source regarding best practices. This can guide how integration can be done in a manner that ensures integration strategies do not fail the expectations of all stakeholders involved.

Further improvements in the response to psychiatric emergencies could include integrating psychiatric care into emergency services. The amount of work established by the systematic reviews has shown an increase in rates of access at EDs, extended lengths of hospital stay, and improved outcomes for patients with the use of CCMs, C-L teams, and telepsychiatry, especially in the most socioeconomically deprived regions. However, much work lies ahead before implementation, considering the significant obstacles inherent in the situation. Lack of clear guidelines, scarcity of resources, and the need to retrain the personnel more effectively pose some of the problems. Further research should consider establishing these aspects and developing higher-capacity treatment models that could easily be implemented in different healthcare settings. This paper will analyze the current perspectives on how mental crisis care could be managed more objectively and efficiently in EDs worldwide and narrow the focus down to the United States. The integration of psychiatric treatment into emergency services is, therefore, essential in the better management of mental crises.

This systematic review discusses CCMs, CL teams, and telepsychiatry regarding their effectiveness in increasing access to psychiatric treatment in EDs, shortening hospital admission stays, and improving overall patient outcomes, especially in disadvantaged areas. However, before deployment, many difficulties must be surmounted. Its limitations are a lack of clearly defined protocols, limited resources, and the staff needs more training. Future research is required to eliminate these barriers and develop new treatment models. Further, these models must be very comprehensive for a wide range of healthcare settings. The purpose of this research is to study some of the strategies for improving management related to mental crises in EDs worldwide, particularly in the United States.

## Conclusions

Incorporating psychiatric care within emergency services is essential to optimizing the management of psychiatric emergencies. Systematic evaluations of the included studies indicate that CCMs, C-L teams, and telepsychiatry have been proven to boost access to EDs, lengths of hospital stay, and patient outcomes, particularly in socioeconomically disadvantaged areas. Nonetheless, substantial challenges must be addressed before deployment. The issues encompass the absence of established protocols, resource constraints, and the necessity for enhanced staff training. Future research must prioritize resolving these issues and creating superior treatment models that can be adapted to diverse healthcare environments.
